# “Resolving the SCD1-oleic acid paradox: majority of oleic acid is converted to free cholesterol in colorectal cancer cells”

**DOI:** 10.1186/s12935-026-04190-w

**Published:** 2026-01-24

**Authors:** Aleksandra Czumaj, Jarosław Kobiela, Adriana Mika, Tomasz Sledzinski

**Affiliations:** 1 Department of Pharmaceutical Biochemistry, Faculty of Pharmacy, Medical University of Gdansk, Gdansk, 80-211 Poland; 2 Department of Surgical Oncology, Transplant Surgery and General Surgery, Faculty of Medicine, Medical University of Gdansk, Gdansk, 80-211 Poland; 3 Department of Environmental Analytics, Faculty of Chemistry, University of Gdansk, Wita Stwosza 63, Gdansk, 80-308 Poland

**Keywords:** Colorectal cancer, Oleic acid, Fatty acid metabolism, SCD1, Free cholesterol

## Abstract

**Objective:**

This study aimed to elucidate the mystery of “disappearing” oleic acid (OA) in colorectal cancer (CRC) cells. The primary objective was to verify if OA is predominantly used as an energy source or as a substrate for the synthesis of other molecules, given the paradox of elevated stearoyl-CoA desaturase-1 (SCD1) expression and lower OA levels in CRC tissue.

**Methods:**

Tissue samples from CRC patients were analyzed for fatty acid (FA) profiles (GC-MS) and SCD1 mRNA levels (real-time PCR). For in-vitro experiments, CRC cell lines (HT-29, WiDr) and a normal human colon cell line (CCD-881-CoN) were incubated with either ^13^C-stearic acid (SA) or ^3^H-OA. GC-MS was employed to track ^13^C-labeled FA conversions, whereas thin-layer chromatography (TLC) followed by radioactivity measurement for ^3^H-labeled metabolites was utilized.

**Results:**

Tracking ^13^C-SA metabolism revealed its conversion to ^13^C-OA but also a significant “disappearance” of its metabolites from the FA pool. Experiments with ^3^H-OA showed that free cholesterol (FCH) was the most abundant molecule originating from ^3^H-OA (67–77% of redirected tritium) in CRC cells, indicating its major metabolic fate.

**Conclusions:**

This study provides strong evidence that in CRC cells, despite SCD1 overexpression, a significant amounts of OA are converted to FCH.

**Supplementary Information:**

The online version contains supplementary material available at 10.1186/s12935-026-04190-w.

## Introduction

The process of carcinogenesis is associated with significant changes in cell metabolism, including fatty acids (FAs) metabolism [1]. Due to FAs significance in cellular metabolism, particularly in energy generation (ATP synthesis), cell proliferation (building blocks for complex lipids in cellular membranes), and metabolism regulation (precursors for signalling molecules, allosteric regulators, substrates for protein acylation, and ligands for transcription factors), FAs are essential compounds for the maintenance and development of rapidly dividing colorectal cancer (CRC) cells [2, 3].

Oleic acid (18:1, OA) is one of the most abundant monounsaturated FA (MUFA) in human cells and blood, as it is a major component of triacylglycerols (TAG) [4, 5]. OA can be provided with the diet or synthesized in cells from palmitic acid (16:0) – a major product of FA synthase (FASN). Palmitate is elongated by FA elongase 6 (ELOVL6) to stearic acid (18:0, SA) and then, 18:0 is desaturated by the activity of stearoyl-CoA desaturase-1 (SCD1) to OA. Research indicates that OA plays a significant role in the context of CRC. Some clinical investigations, including our previous research, showed an elevation of OA concentration in the plasma of CRC patients with no difference in diet intake [6–9]. Surprisingly, despite elevated expression of SCD1, the level of OA in the cancer tissue of CRC patients is significantly lower compared to normal colon tissue [6, 10–12]. Based on our previous in-vitro experiments [13], the release of OA from CRC cells may partially contribute to this difference, but doesn’t explain it in full, and suggests the metabolism of OA into other metabolites than FAs or its utilization in β-oxidation and the Krebs cycle as a substrate for cellular energy production in CRC cells. It should be noted that cancer cells have high energy demands due to their rapid proliferation activity [14, 15]. Thus, in the present study, we aimed to resolve the mystery of the “disappearing” OA in CRC cells. The objective of the present study was to verify, using radiolabelled FAs, if OA is used as an energy source or as a substrate for the synthesis of other molecules in CRC cells.

## Materials & methods

### Tissue samples from CRC patients

The study included tumor grades 1–3, along with normal large intestinal mucosa tissue samples from 26 CRC patients treated with surgical resection of the large bowel segment. None of the patients received preoperative neoadjuvant treatment. Patients characteristics is presented in Supplementary Table 1. Following collection, the samples were frozen in liquid nitrogen and subsequently stored at −80 °C until FA profile analysis (GC-MS) and mRNA levels analysis (real-time PCR). The protocol of the study was compliant with the Declaration of Helsinki of the World Medical Association and with approval from the Local Bioethics Committee at the Medical University of Gdansk (decision no. NKBN/487/2015). Written informed consent was obtained from all the patients prior to the study.

### Cell culture & treatment

One month prior to initiating the study, the HT-29, WiDr, and CCD-881-CoN cell lines were obtained from the American Type Culture Collection (ATCC, Manassas, VA, USA). HT-29 and WiDr cell lines were utilized as the colorectal adenocarcinoma model and CCD-841-CoN was selected as a normal human colon model. All cell lines were cultured at a temperature of 37 °C in 5% CO_2_ environment, and all experiments were performed on cells between passages 3 and 8. At the beginning and at the end of the study the cell lines have been tested for mycoplasma contamination.

Two different types of culture media were used in the study: base medium and two experimental media. The base medium comprised the media recommended by the cells supplier (McCoy’s 5 A Medium for HT-29, Eagle’s Minimum Essential Medium for WiDr and CCD-881-CoN) supplemented with 10% foetal bovine serum, 100 units/ml penicillin and 100 µg/ml streptomycin. The experimental medium A was prepared by combining the base medium and SA labelled with ^13^C (^13^CH_3_ (^13^CH_2_)_16_^13^CO_2_H). The experimental medium B was formulated with the base medium and OA labelled with tritium ([9,10-^3^H(N)]-OA).

Cells were seeded on 100 mm plates at 0.3 × 10^6^ density and after 24 h incubated with 75µM ^13^C-SA or 50nM ^3^H-OA for 72 h. To confirm that the experimental concentration of ^3^H-OA and ^13^C-SA did not have any effect on cell viability the Trypan Blue staining was performed and cell viability was measured using the Countess 3 Automated Cell Counter (ThermoFisher Scientific, Waltham, MA, USA). We did not find any significant effect of radiolabeled SA nor OA in used concentrations on cell viability. All cell culture reagents and ^13^C-SA were obtained from Sigma-Aldrich (Saint Louis, MO, USA). ^3^H-OA was obtained from Perkin Elmer (Boston, MA, USA).

### The analysis of mRNA levels by real-time PCR

Following the manufacturer’s instructions, the RNeasy Plus Universal Kit (Qiagen, Hilden, Germany) was used to isolate total RNA from CRC and normal tissue samples. The concentration and purity of the RNA were assessed using a NanoDrop One spectrophotometer (ThermoFisher Scientific). The quality and integrity of the RNA was assessed by Experion capillary electrophoresis (Bio-Rad, Hercules, CA, USA). Only samples with RQI over 7.0 were used for the analysis. To get rid of any DNA contaminations, all samples were DNase-treated (DNase I, RNase-free, ThermoFisher Scientific). Using RevertAid First Strand cDNA Synthesis Kit (ThermoFisher Scientific) and random hexamer primers, 1 µg of total RNA was converted into first-strand cDNA for PCR amplification. The CFX Connect Real-Time PCR Detection System (Bio-Rad, Hercules, CA, USA) was used for PCR amplification. The stearoyl-CoA desaturase 1 (SCD1) gene expression was determined using the delta-delta Ct technique. For standardization, the β-actin gene was used.

### GS/MS analysis of fatty acids

Total lipid extraction from patient tissues was performed according to the method of Folch et al. [16]. Lipids were then hydrolyzed with KOH in methanol and washed with a mixture of water and n-hexane. The n-hexane was evaporated to dryness under a nitrogen stream. Free FAs were methylated with 10% boron trifluoride solution. FA methyl esters were analyzed using a GC-EI-MS QP-2010 SE (Shimadzu, Japan), as in our previous studies [17]. In addition, the conversion products of ^13^C-labeled stearic acid in HT-29 cells were analyzed using the same GC-EI-MS in selective ion monitoring (SIM) mode. Characteristic fragment ions for saturated and monounsaturated FA, which can be formed in cells from SA, were used for identification. Polyunsaturated FAs cannot be produced in human cells from SA [18]. Total analysis time was 67.5 min, and the column temperature was set in the range of 60–310 °C. To achieve calibration linearity, least squares regression analysis was used, comparing peak area ratios to increasing concentrations of standards.

### Thin-layer chromatography (TLC) and radioactivity measurement

After incubation with ^3^H-OA cell plates were washed twice with Phosphate-buffered saline (PBS) and then scraped in PBS and collected in centrifuge tubes. The cell pellets were obtained by centrifugation at 4 °C at 1,000×g for 5 min and resuspended in 25µL PBS. Lipid extraction was performed with a Lipid Extraction Kit (Abcam, Cambridge, EN) according to manufacturer instructions. In brief, 500µL of the Lipid Extraction Buffer was added to each sample. Then samples were vortexed for 2 min, agitated for 20 min on an orbital shaker at room temperature, and centrifuged at 10,000 x g for 5 min. The supernatant was collected and dried at 37 °C overnight in a dry incubator (Hybridiser HB-1D, Techne, Somerset, EN). The lipid extract was resuspended in a Suspension Buffer. The aqueous phases were transferred in other tubes.

Each lipid extract was split into two samples for polar and non-polar lipid separation. Lipid classes were separated on silica gel 60 with concentration zone aluminium sheets (Sigma-Aldrich). Mobile phases were chloroform-methanol-water (65:25:4) for separation of the polar lipid and hexane-diethyl ether-acetic acid (90:10:1) for the non-polar lipids. Samples were loaded on the TLC sheet together with reference solutions: Polar Lipid Mixture and Non-Polar Lipid Mixture B (Cayman Chemical, Ann Arbor, MI, USA). TLC was carried out at room temperature and the plates were allowed to develop up till the solvent reached a distance of about 1 cm from the top of the plate.

The crystalline iodine was sublimated into the gas phase in the staining chamber and equilibrated overnight so that the iodine vapor filled the entire glass tank. Then plates were placed in iodine-containing chamber for 15 min, and all TLC spots were marked with a pencil for further analysis.

From each TLC spot lipids were recovered. Marked spots were scraped off silica with razor blades in glass vials and chloroform-methanol (2:1) was added. Vials were incubated for 30 min with gentle shaking, centrifuged for 3 min at 4,000 x g, and the supernatant was transferred into a new glass vial. The organic phase was obtained after adding 0.9% NaCl and solvents were evaporated under a stream of nitrogen. Polar lipid fractions were dissolved in chloroform–methanol (2:1) and non-polar fractions were dissolved in chloroform.

The collected lipid fractions and medium samples were transferred to scintillation vials and filled with Ultima Gold F and Ultima Gold scintillation cocktail (Perkin Elmer), respectively. After rigorously mixing, radioactivity was measured with a Beckman LS 6000IC liquid scintillation counter (Beckman Coulter). The values were normalized to cell numbers (lipid fractions) or sample volume (media samples).

### Statistical analysis

Three independent cell culture experiments in duplicates were performed for all assays. All data were presented as mean ± SD. Statistical analysis of data was performed with Statistica 13 software (TIBCO Software Inc., Palo Alto, CA, USA). The Shapiro-Wilk test checked the normality of analysed parameters. For comparison between two groups t-student test was used, for multiple group comparison ANOVA followed by post-hoc Scheffe test was used. Results with *p* < 0.05 were considered to be statistically significant. Before the start of the experiment, the required minimum number of tissue samples from patients (power = 0.8) has been estimated by the “A priori required sample size” test by G*Power Software 3.1.9.7 (Manchester Metropolitan University) [19]. For all post hoc analysis of results of cell treatment by radiolabeled FA: compute achieved power calculations were carried out using G*Power 3.1.9.7. All analyzed result reached the threshold power (1-β err prob) ≥ 0.80. For all cell culture experiments the investigation was executed across three independent experimental replications. Within each replication, three independent biological replicates per cell line was used. Data acquisition from each independent biological replicate was conducted in technical duplicate, yielding a total of 18 individual measurements per cell line across all analysis, or 9 independent samples per cell line.

## Results

### SCD1 expression and OA levels in CRC tissue and cells

**Fig. 1:**
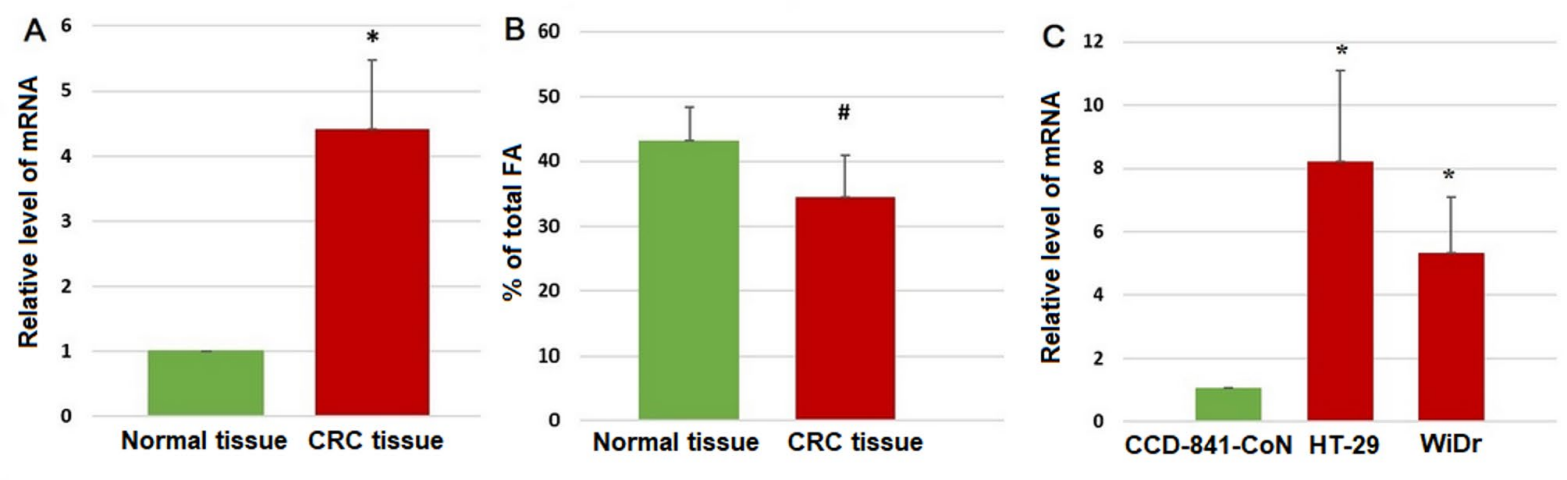
SCD1 mRNA level (**A**) and oleic acid level (presented as a % of total FA) (**B**) in normal colon mucosa and CRC tissue; SCD1 mRNA level (**C**) in normal colon cells (CCD-841-CoN) and in CRC cells (HT-29, WiDr). Data presented as mean ± SD. * *p* < 0.05; # *p* < 0.01.(Some of these data were used in our previous paper [20])

In cancer tissue of CRC patients, the SCD1 was overexpressed whereas OA levels were lower comparing to normal colon mucosa (Fig. 1A and B), as we also presented in our previous studies [6, 20]. As we are not able to track the transformations of FAs in patients body we decided to perform further experiments in in-vitro model. We’ve chosen CRC cells (HT-29, WiDr) and normal colon cells (CCD-841-CoN) for this experiments. First, to verify if those cells reflected SCD1 overexpression observed in CRC patients the mRNA levels of SCD1 were measured. Those experiments confirmed the SCD1 overexpression seen in patients (Fig. 1C).

### Fate of ^13^C-stearic acid in CRC cells

**Fig. 2:**
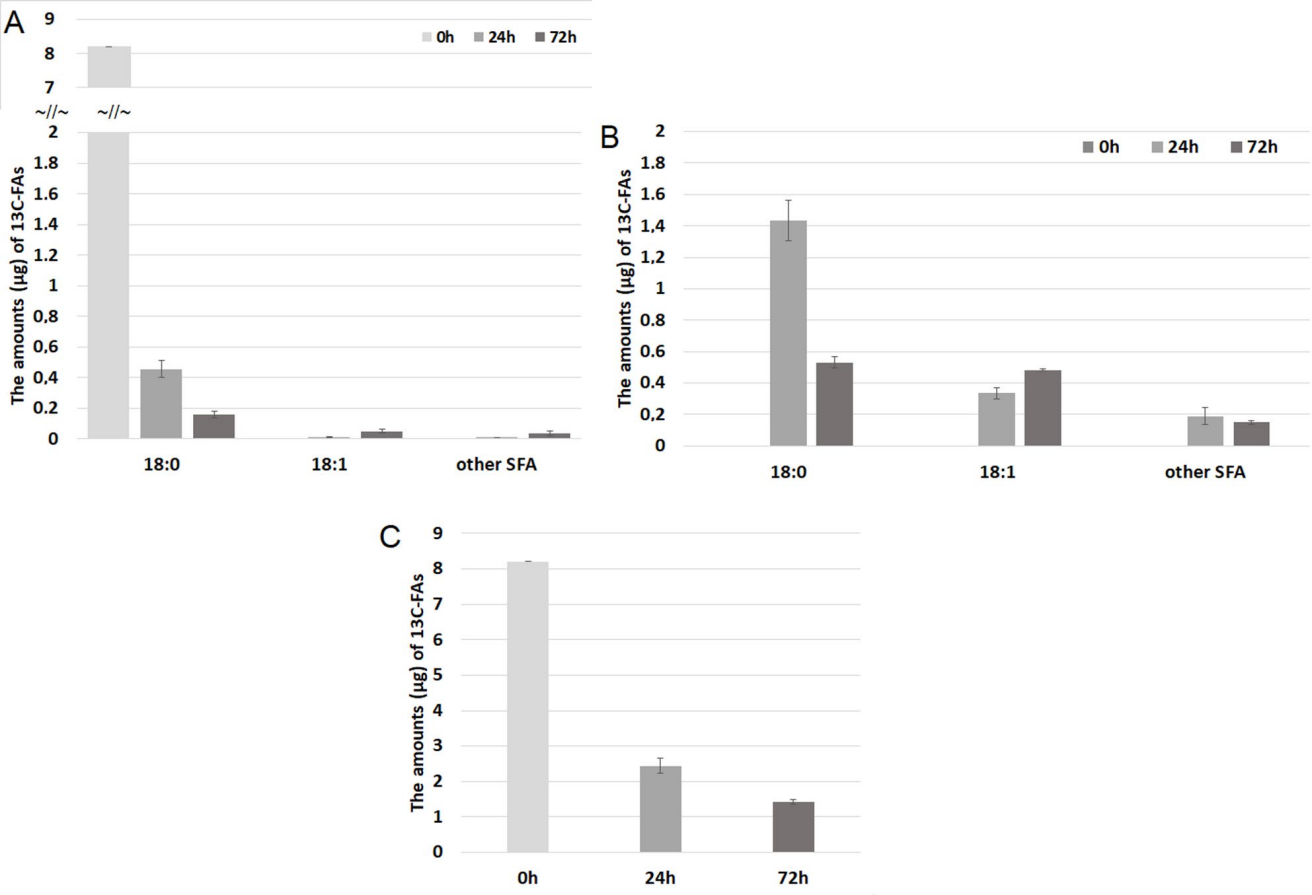
The amounts (µg) of detected ^13^C-FAs during 72 h incubation in (**A**) cell culture medium, (**B**) HT-29 cells, and (**C**) total ^13^C-FAs (from cells and medium). (Some of these data were used in our previous paper [13])

In the next step we have tracked the conversions of radiolabeled SA to OA, and potentially to other FAs. ^13^C-SA has been added into the culture medium of HT-29 CRC cells, and its level was monitored after 24 h and 72 h in both cells and culture media by GC-MS in SIM mode. We have found that ^13^C-SA entered the cells and it was mainly converted into ^13^C-OA, and into the lesser extent to other ^13^C-saturated FA with very long chains (20–26 carbon atoms) (compare Fig. 2A and B). Moreover, some small amounts of ^13^C-OA were released by CRC cells into the culture media (Fig. 2A). An exemplary chromatograms and mass spectra of ^13^C-SA and ^13^C-OA are presented in Supplementary Fig. 1. Most importantly, the total ^13^C-FAs in both cells and culture media decreased during the incubation (Fig. 2C).

These results suggest that (a) stearic acid is mostly converted into OA in CRC cells, probably due to SCD1 overexpression; (b) most of OA is than converted into metabolites other than FAs, that cannot be detected by our GC-MS method. Considering, that before GC-MS analysis we have performed lipid hydrolysis, all FAs were measured, both free FAs (FFA) and those esterified in complex lipids like TAG, phospholipids, sphingolipids and cholesterol esters. Taking the above results into account we hypothesized that OA can be transformed to acetyl-CoA by β-oxidation and then used for ATP production by total utilization in Krebs Cycle, or alternatively acetyl-CoA can be converted into other metabolites like cholesterol or ketone bodies. The potential metabolites, which could originate from OA in CRC cells may have different chemical properties, including water solubility so we decided to use another analytical approach for further experiments.

### Uptake and distribution of ^3^H-oleic acid

We have incubated the cells in the presence of tritium radiolabelled OA. In this approach both lipids and hydrophilic metabolites of OA can be detected by liquid scintillation counter. In these experiments we used two CRC cell lines and additionally normal colon mucosa cells to better evaluate the characteristics of OA metabolism in CRC cells.

**Fig. 3:**
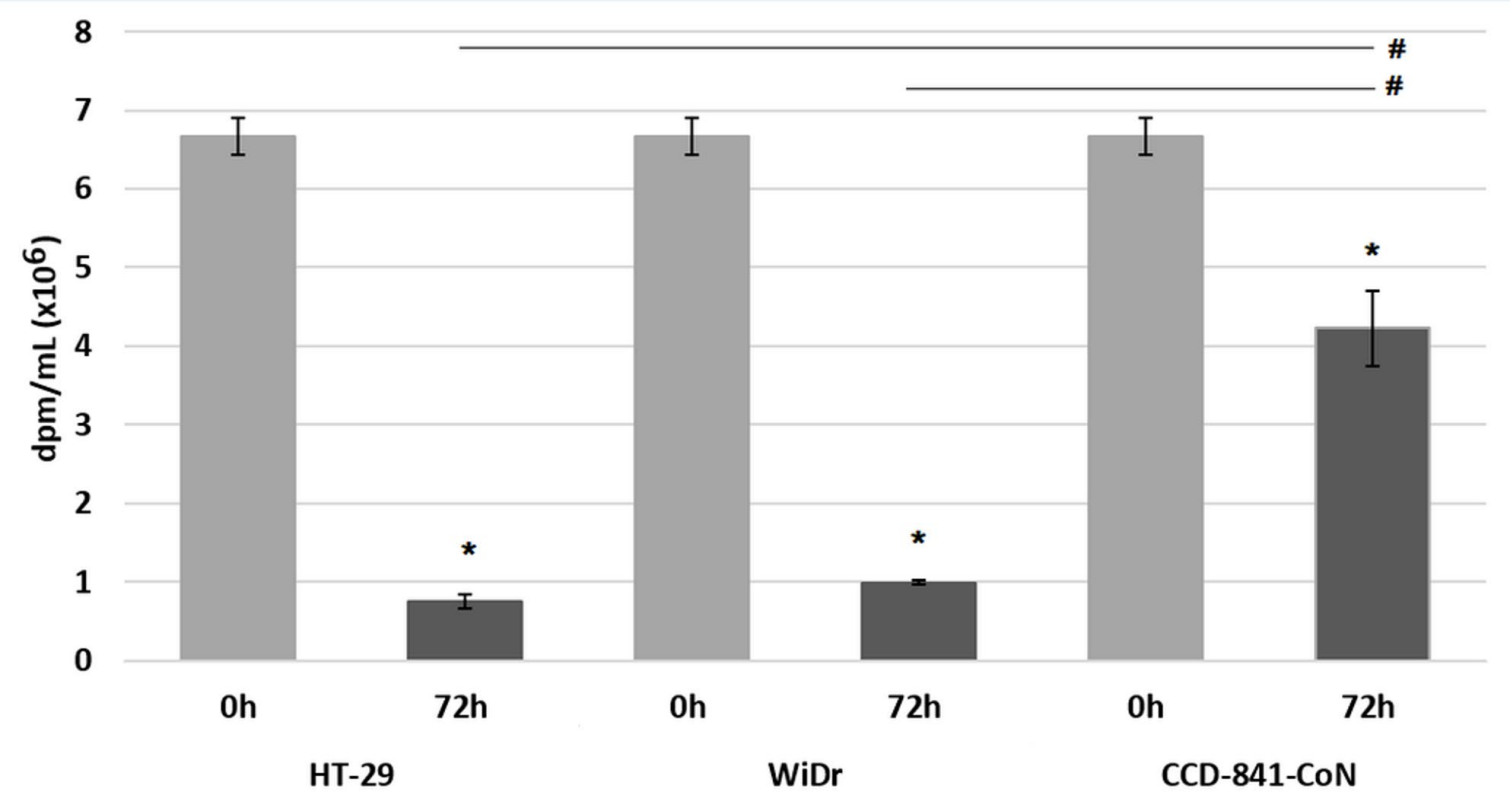
Total radioactivity (dpm/mL) in cell culture media at the 0 h and 72 h incubation of colon cancer cells (HT-29, WiDr) and normal colon cells (CCD-841-CoN) with [9,10-^3^H(N)]-oleic acid. Data presented as mean ± SD. * *p* < 0.05 in comparison to base medium at 0 h, # *p* < 0.05 in comparison to normal colon cells, dpm - disintegrations per minute

First, we investigated the rate of radiolabelled OA uptake by CRC cells (HT-29, WiDr) and normal colon cells (CCD-841-CoN) by measuring radioactivity in culture media. After 72 h of incubation with [9,10-^3^H(N)]-OA, CRC cells have up-taken more than 85% of labelled OA from the media whereas normal colon cells have up-taken only 36% (Fig. 3). The difference observed between normal and CRC cells was statistically significant.

**Fig. 4:**
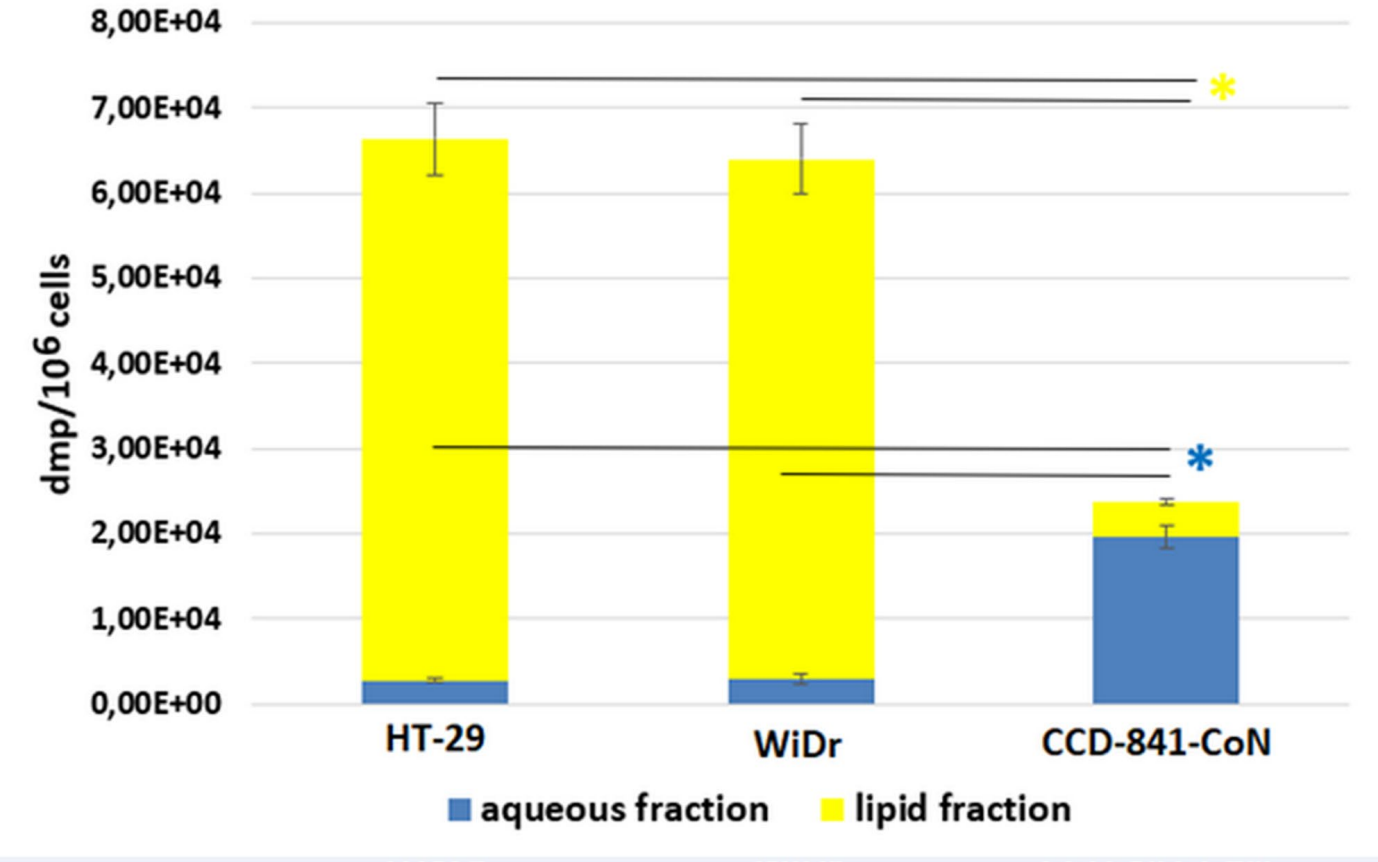
Total radioactivity (dpm/10^6^ cells) in aqueous and lipid cells fractions after 72 h incubation with [9,10-^3^H(N)]-oleic acid in colon cancer cells (HT-29, WiDr) and normal colon cells (CCD-841-CoN). Data presented as mean ± SD. * *p* < 0.05 in comparison to normal colon cells, dpm - disintegrations per minute

To evaluate whether the OA will be converted to lipid molecules or water soluble molecules, the total tritium level was measured in the aqueous and lipid fractions of the cells. Data analysis revealed that in CRC cells around 95% of tritium signal was detected in the lipid fraction, whereas only 17% of tritium signal came from lipid fraction in normal colon cells (Fig. 4).

### Identification of ^3^H-OA derived Polar and non-polar lipids

**Fig. 5:**
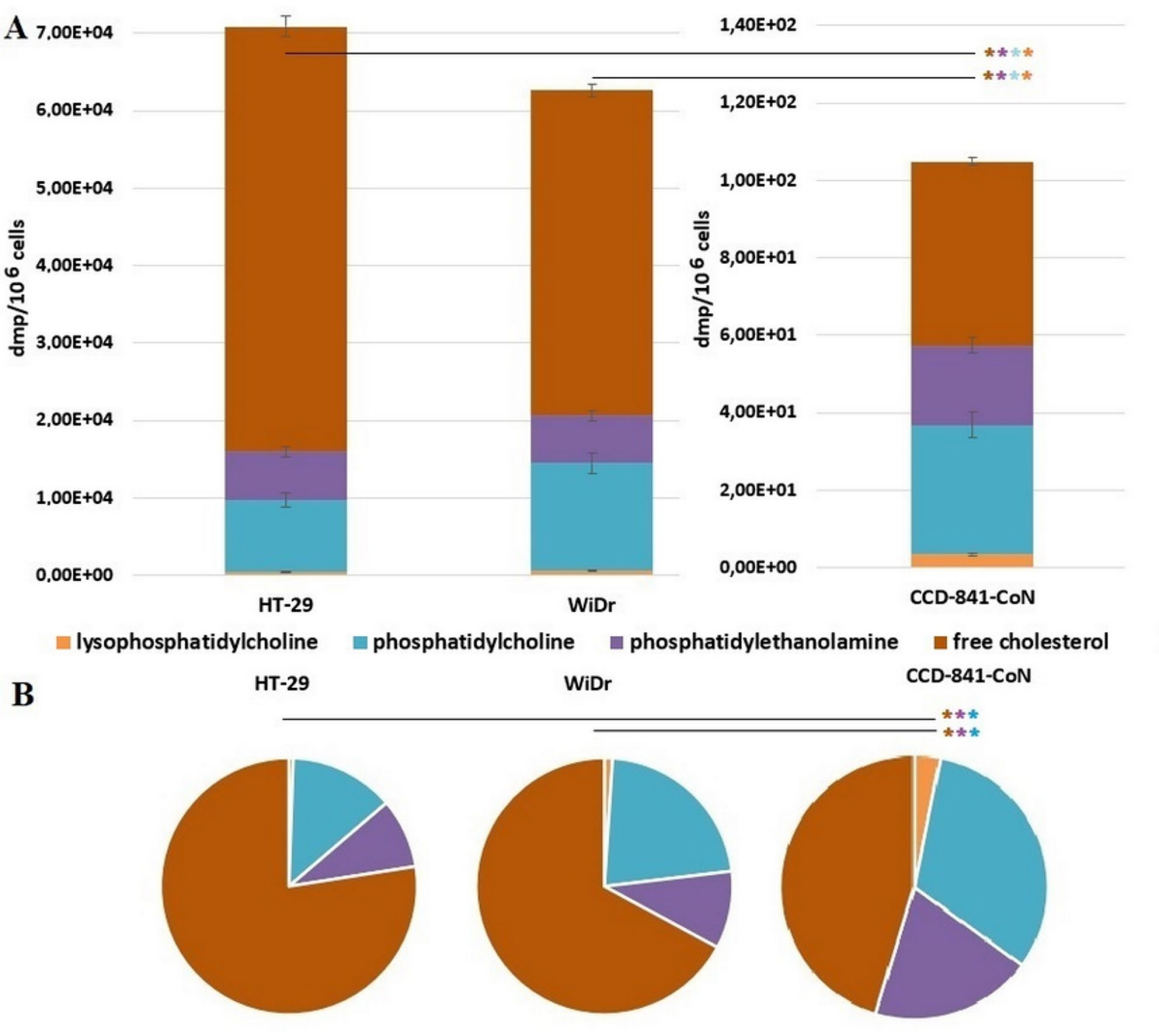
**A**) Total radioactivity (dpm/10^6^ cells) in polar lipid fractions after 72 h incubation with [9,10-^3^H(N)]-oleic acid in colon cancer cells (HT-29, WiDr) and normal colon cells (CCD-841-CoN). Data presented as mean ± SD. **B**) % of the total radioactivity of each polar lipid fraction after 72 h incubation with [9,10-^3^H(N)]-oleic acid in colon cancer cells (HT-29, WiDr) and normal colon cells (CCD-841-CoN). * *p* < 0.05 in comparison to normal colon cells, dpm - disintegrations per minute

The presence of tritium in lipid fraction is an evidence that exogenous OA is present in cells and/or it was used for other lipid synthesis. The subsequent efforts focused on identifying polar and non-polar lipids, containing tritium from OA. By TLC method we were able to separate polar lipid fraction containing lysophosphatidylcholine (LPC), phosphatidylcholine (PC), phosphatidylethanolamine (PE), and free cholesterol (FCH) (Fig. 5A, B).

In all types of examined cells FCH was the most abundant lipid detected by our method in polar lipid fraction, whereas LPC was least abundant. Moreover, there was no significant difference in the % of the total radioactivity of LPC between studied types of cells. The significant difference in % of the total radioactivity of polar lipid fractions between normal and CRC cells was observed for FCH, PC, and PE. The FCH fraction contained 45% of tritium from ^3^H-OA redirected to polar lipid synthesis in normal colon cells, whereas CRC cells incorporated 77% and 67% (HT-29 and WiDr, respectively) tritium in FCH among polar lipids. In normal colon cells 32% of up taken ^3^H-OA redirected to polar lipid synthesis was used for PC formation, whereas in CRC cells it was 13% and 22% (for HT-29 and WiDr, respectively). The PE synthesis used 20% of up-taken ^3^H-OA redirected to polar lipid synthesis in normal colon cells, whereas CRC cells used 9% and 10% (HT-29 and WiDr, respectively).

**Fig. 6:**
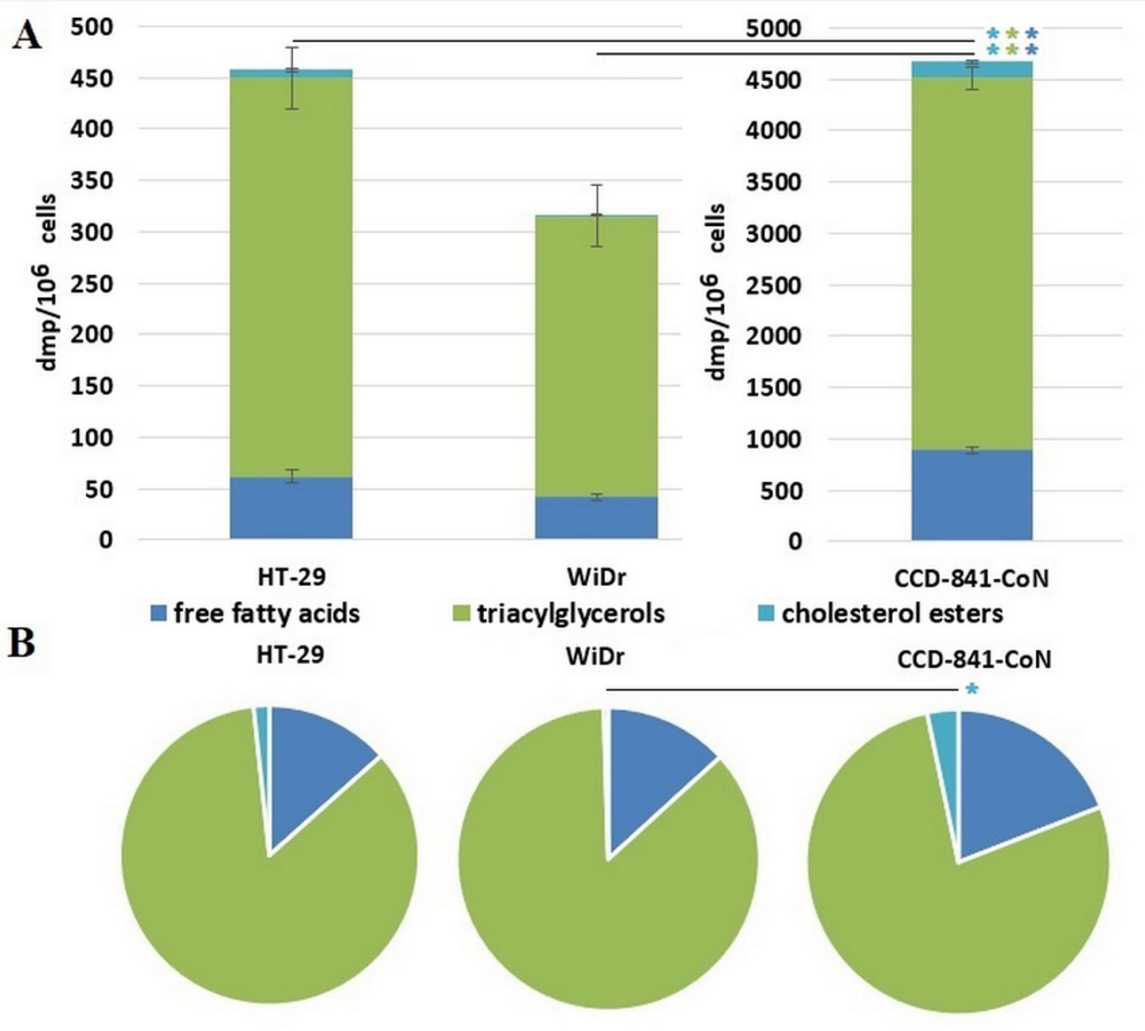
**A**) Total radioactivity (dpm/10^6^ cells) in nonpolar lipid fractions after 72 h incubation with [9,10-^3^H(N)]-oleic acid in colon cancer cells (HT-29, WiDr) and normal colon cells (CCD-841-CoN). Data presented as mean ± SD. **B**) % of the total radioactivity of each lipid fraction after 72 h incubation with [9,10-^3^H(N)]-oleic acid in colon cancer cells (HT-29, WiDr) and normal colon cells (CCD-841-CoN). * *p* < 0.05 in comparison to normal colon cells, dpm - disintegrations per minute

Among nonpolar lipids, FFA, cholesterol esters (CHE), and TAG were detected by our method. In all types of cells TAG fraction was the most abundant nonpolar lipid fraction containing tritium originating from exogenous radiolabeled OA (Fig. 6A, B).

In normal colon cells 77% of up taken ^3^H-OA redirected to nonpolar lipid synthesis was used for TAG formation, whereas in CRC cells it was 85% and 86% (for HT-29 and WiDr, respectively). The CHE was the least abundant nonpolar fraction containing tritium in all types of cells. However, in normal colon cells the 3% of up taken ^3^H-OA redirected to CHE synthesis than inwas greater than in CRC cells (1% in HT-29 and 0.5% in WiDr). The % of the total radioactivity of FFA did not differ significantly between studied types of cells.

## Discussion

This study provides novel evidence that OA in CRC cells is predominantly converted to FCH. The impetus for this research arose from a paradoxical observation: the overexpression of SCD1 in CRC tissue was accompanied by concomitant lower levels of SCD1 product – OA. OA metabolism may plays a significant role in proliferation, migration, and metastasis of CRC. For example, in KRAS/p53-mutant CRC, OA induces metastasis by activating the ANGPTL4/IL-8/NOX4 axis and KRAS signaling, independent of EGFR, facilitating epithelial-mesenchymal transition (EMT) and metastasis [21]. The studies providing better insight into OA metabolism in cancer can offer new therapeutic targets. Previous studies have also documented the overexpression of SCD1 in CRC and other malignancies [20, 22–26]. Upregulated SCD1 may promote metastasis, proliferation, EMT, and is associated with poor prognosis, particularly under high-glucose conditions [12]. SCD1 drives CRC metastasis by suppressing PTEN, enhancing migration/invasion via ChREBP activation in hyperglycemia. Inhibition of SCD1 impairs lipid droplet formation and triacylglycerol accumulation, activating AMPK-mediated lipolysis/lipophagy, that enables peritoneal metastasis through CAF-derived lipids compensating low SCD1. High SCD1 expression correlates with aggressiveness and reduced survival in CRC patients [12, 27–29]. By contrast to SCD1 overexpression, a lower level of OA in CRC was reported in our previous papers [6, 30] and similar results were published by other authors [31]. Notably, lower OA in CRC tissue is associated with lower TAG levels [32], of which OA is the main component [4, 5]. Given the limitations of conducting metabolite tracking studies within a patient’s body, to resolve this mystery, we first tracked ^13^C-SA in the in-vitro model. The experiment with tracking of ^13^C-SA conversion to ^13^C-OA confirmed high activity of SCD1 in CRC cells. Crucially, the total ^13^C-FAs decreased during incubation, despite the fact that lipid hydrolysis was performed before GC-MS analysis, meaning that all FAs (free and those included in TAG, phospholipids, cholesterol esters etc.) were measured. This “disappearance” strongly indicated that FAs were being transformed into metabolites other than FAs. To differentiate these metabolic fates, we used ^3^H-OA tracking.

The radioactivity in the aqueous fraction was much lower than in the lipid fraction in CRC cells, whereas higher in normal colon cells. ^3^H in the aqueous fraction may represent ^3^H_2_O produced during the complete oxidation of OA. This finding suggests that the majority of OA is directed towards other lipid synthesis, potentially limiting the portion of OA fully oxidized for ATP production in CRC cells compared to normal colon cells. This is consistent with the assumption that cancer cells prefer glucose as an energy substrate and metabolize it to a large extent to lactate (Warburg effect) [33]. Another possible metabolites containing ^3^H, that may be found in the aqueous fraction, are water-soluble molecules produced from OA, precisely, from acetyl-Co which is formed by β-oxidation of OA. These may include carboxyl acids, amino acids, or ketone bodies (KB). It is important to state that KBs are not universally required by CRC cells for energy or survival, and in fact, the dominant evidence suggests that the main ketone body, β-hydroxybutyrate (BHB), may exert anti-proliferative and anti-tumorigenic effects on CRC cells [34]. Nevertheless, in CRC cells only a small amount of radioactivity has been found in the aqueous fraction.

In turn, the lipid metabolites, which constitute a majority of radiolabeled OA derivatives have been studied in more detail. Among polar lipids produced from radiolabeled OA in CRC cells, the vast majority was a FCH. The amounts of radiolabeled FCH produced from OA in CRC cells were higher than in normal colon cells. The fact that large amounts of OA are converted into FCH in CRC cells suggests the importance of this metabolite for CRC cell function. The transformation of OA to FCH require the process of β-oxidation to acetyl-CoA, which is than transformed into cholesterol. Thus, we can expect that β-oxidation is active in CRC cells. However, it must be noted that the absolute level or specific activity of β-oxidation was not directly quantified in this study, and this conclusion is thus inferential, based solely on the tritium signal distribution. This massive synthesis of FCH is consistent with the known essential role of FCH as a building block for cell membranes in rapidly proliferating cancer cells. It should be also noted, that in our previous study, we have found higher levels of FCH and increased expression of HMG-CoA reductase in CRC tissue than in normal colon tissue [30]. The most possible pathway of conversion of OA into FCH in CRC cells is first OA β-oxidation to acetyl-CoA and then synthesis of cholesterol from acetyl-CoA. Acetyl-CoA, which is a key metabolite in these conversions, may also partly represent the radioactivity found in the aqueous fraction. It should also be pointed out that β-oxidation of OA to acetyl-CoA provides NADH and FADH_2_, which can be used for ATP synthesis in mitochondria. This additional portion of energy produced during the conversion of OA to cholesterol is highly desirable in cancer cells. FCH plays a significant, multifaceted role in cancer, supporting tumor cell survival, growth, and progression through various mechanisms. FCH accumulates both within cancer cells and the tumor microenvironment (TME), acting as a central driver of cancer pathogenesis [35–37]. In the TME, FCH drives metabolic reprogramming, oncogenic signaling, membrane remodeling, and immune suppression. As a key structural component, FCH particularly enriches cell membranes’ lipid rafts. These rafts are not merely structural elements but critical platforms for signaling pathways that promote cancer cell proliferation, migration, and invasion. By facilitating pathways such as PI3K/AKT, MAPK, and SREBP, FCH consequently boosts cell survival and metastatic potential [38, 39]. Moreover, the high cholesterol levels within the TME significantly impair anti-tumor immunity. They induce NK/CD8 + T cell dysfunction via mitochondrial impairment and promote T cell exhaustion through the elevation of cholesteryl esters. FCH also contributes to the cancer progression by promoting angiogenesis in hypercholesterolemic models and disrupting anti-tumor signaling by altering membrane fluidity [40, 41].

Another polar lipids containing ^3^H from radiolabeled OA were phospholipids. In these metabolites, OA may be a major component, and it should be stressed that this is the major FA which presence was confirmed in experiment with the FAs labelled by ^13^C. The detection of synthesized phospholipids, alongside FCH, is consistent with the finding that CRC cells favor the synthesis of polar lipids, which are crucial for forming and maintaining cell membranes.

**Fig. 7:**
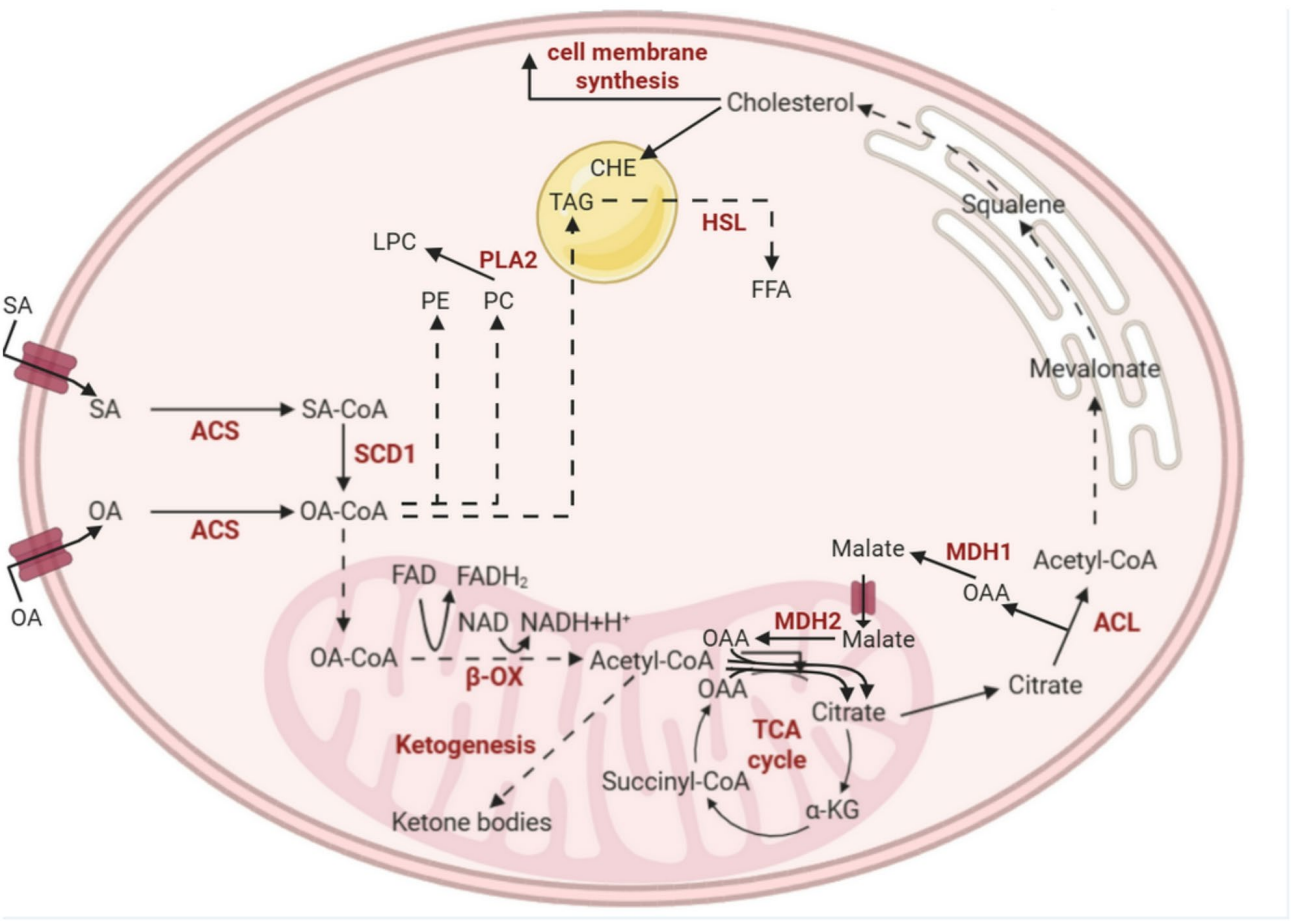
The potential pathways of conversion of oleic acid into different metabolites in CRC cells. α-KG – alpha ketoglutarate, β-OX- β-oxidation, ACL - ATP citrate lyase, ACS - acyl-CoA synthetases, FFA – free fatty acid, HSL - hormone-sensitive lipase, LPC – lysophosphatidylcholine, MDH1 - Malate dehydrogenase 1, MDH2 - Malate dehydrogenase 2, OA – oleic acid, OAA - oxaloacetic acid, PLA2 - phospholipase A2, PC – phosphatidylcholine, PE – phosphatidylethanolamine, SA - stearic acid, SCD1 - stearoyl-CoA desaturase 1, TAG – triacylglycerol, TCA Cycle - Krebs cycle

By contrast, the majority of nonpolar lipids containing ^3^H were TAGs, which can also contain radiolabeled OA. There is also a possibility of β-oxidation of ^13^C-OA into acetyl-CoA, and then, ^13^C carbon atoms may be used for *de novo* SFA and other MUFA synthesis – a process that is also upregulated in CRC cells. These newly synthesized FAs may potentially be included in TAG or phospholipids, however, our experiment with ^13^C-labelled SA showed that most of the pool of FA containing ^13^C originating from SA is OA. The potential pathways of conversion of OA into different metabolites, based on the results presented in this study are summarized in Fig. 7.

Comparing the overall magnitude of the dpm signals, our data have shown that the synthesis of polar lipids was favored over nonpolar lipids synthesis in CRC cells. The dominance of polar lipids in cell was also observed in the human prostate cancer cell lines (22RV1, LNCaP, and DU145) in comparison to the prostate non-malignant cell line (PNT1a) [42]. Polar lipids are primarily crucial for maintaining the integrity and functionality of cell membranes, influencing cell signaling and immune evasion, whereas nonpolar lipids primarily contribute to the overall energy balance. Studies have shown that increased level of nonpolar lipids lead to their accumulation in the form of lipid droplets, and accompanies the most aggressive and resistant cancer phenotypes [43, 44]. Accumulation of the PC in our CRC cells is in line with previous observations made by our team and other researchers, that CRC tissues have higher levels of this phospholipid than normal colon tissue [32, 45]. Increased PC level can support: accelerated cell growth (by providing cellular membrane component), metabolic flexibility under stress conditions (by providing energy source), cell survival and migration (by PC-derived lipid mediators), and resistance to therapy (by modulation of DNA methylation and DNA repair) [46, 47]. PE is a crucial phospholipid that plays significant roles in cellular processes, including membrane dynamics and signaling pathways. We observed higher level of PE in CRC cells than in normal cells. A higher level of PE was also observed in CRC tissue by Wang et al. [48]. In our previous research we demonstrate lower content of TAG in CRC tissue in comparison to normal tissue [30]. In this study, our in-vitro model shows the same relation. TAG and phosphatidylcholine can form lipid droplets, where TAG serves as the core neutral lipid, and phosphatidylcholine is a major component of the monolayer that coats and stabilizes these droplets. Increased lipid droplet accumulation can confer resistance to chemotherapy in CRC cells (SW620, LoVo, Hct116, Hct8, SW480, HT-29) [49].

Cancer cells may obtain FA from *de novo* lipogenesis and exogenous lipid uptake. Exogenous uptake of FA allows for metabolic flexibility in cancer cells to not only sustain their rapid proliferative rate but also provide an essential energy source during conditions of metabolic stress. In this study, we showed enhanced OA uptake by CRC cells.

Some limitations of this study should be indicated. Firstly, it is important to note, that the HT-29 and WiDr cell lines are characterized by specific driver mutations, including the p53^R273H^ and BRAF^V600E^ gain-of-function alterations. These specific genetic backgrounds are known to profoundly influence metabolic behavior. Consequently, the observed high anabolic shunting phenotype may be genotype-specific and not universally representative of all CRC subtypes. Thus, this study provides conclusion that are based on metabolism in used CRC cell lines and requires further validation. Another limitation is the lack of verification how different glucose concentrations affect OA to FCH conversion. In our experiments, in accordance with the supplier’s recommendations, the HT-29 and WiDr lines were cultured in media with different glucose concentrations (3.0 g/L for HT-29 vs. 1.0 g/L for WiDr). We have found that high OA to FCH conversion was present in both cell lines irrespective of the initial glucose levels (1.0 g/L versus 3.0 g/L). Importantly, the patients data presented in this paper (overexpression of SCD1 and decreased OA in CRC tissue) as well as increased levels of FCH in CRC tissue (presented in our previous paper [11]) suggest that in glucose concentrations persisting in patients CRC tissue the conversion of OA to FCH is active. The limitation of this study is also the fact that the absolute level or specific activity of β-oxidation was not directly quantified in this study, and the conclusions are based solely on the tritium signal distribution. It should be also noted that the direct incorporation of newly synthesized FCH into the plasma membrane was not experimentally demonstrated in this study. The methodological limitation has to be recognized that the identification of FCH relies on its co-migration with a reference standard during TLC separation. While this method strongly indicates the identity based on physical properties, definitive structural confirmation via mass spectrometry was not performed, meaning the conclusion is based on highly correlational, rather than unequivocal, evidence.

## Conclusion

We provide evidence that despite the overexpression of SCD1 and efficient synthesis of OA in CRC cells, a significant portion of this FA is converted into metabolites other than FA. Our experiment with tritiated OA strongly suggests that the majority of this FA is primarily transformed into FCH, with only a small fraction fully oxidized for energy production in CRC cells. These conclusions are based on in vitro results from the specific cell lines tested (HT-29 and WiDr) and require further validation in diverse CRC models.

## Supplementary Information

Supplementary Material 1

## Data Availability

Data will be made available on request.
