# Supplementary material for: “Resolving the SCD1-oleic acid paradox: majority of oleic acid is converted to free cholesterol in colorectal cancer cells”
Source: Cancer Cell Int. 2026 Jan 24;26:94. doi: 10.1186/s12935-026-04190-w (PMC12910776; doi:10.1186/s12935-026-04190-w)
Supplement: Supplementary file 1 — Supplementary Material 1 [file 12935_2026_4190_MOESM1_ESM.docx]

**Supplementary Table 1.** Patients characteristics. BMI – body mass index, T stage – tumor stage, UICC stage - Union for International Cancer Control stage

|  | **N** |
| --- | --- |
| Sex | Male = 19  Female = 7 |
| T stage | T0 = 1  T1 = 1  T2 = 6  T3 = 18 |
| Lymph node status | N0 = 15  N1 = 9  N2 = 2 |
| UICC stage | I = 2  II = 22  III = 2 |
| Tumor localization | Rectosigmoid junction = 7  Rectum = 5  Caecum = 5  Sigmoid colon = 3  Ascending colon = 2  Hepatic flexure = 2  Descending colon = 1  Transverse colon = 1 |
|  | **Mean ± SD** |
| Age (years) | 65,96 ± 12,80 |
| BMI (kg/m^2^) | 27,14 ± 5,09 |


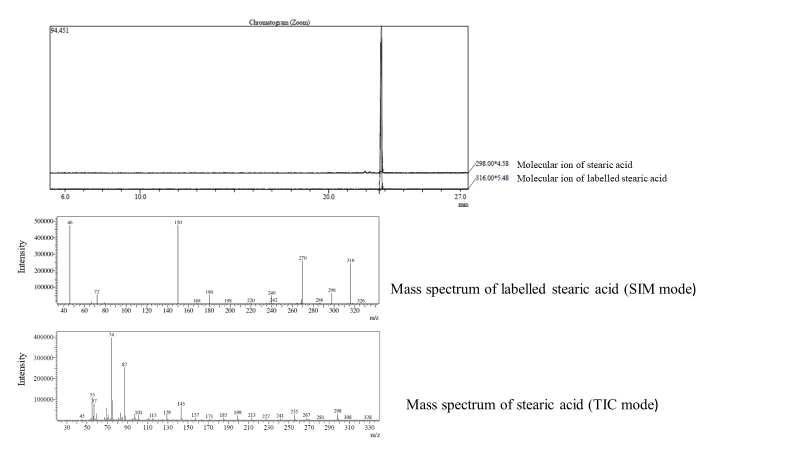

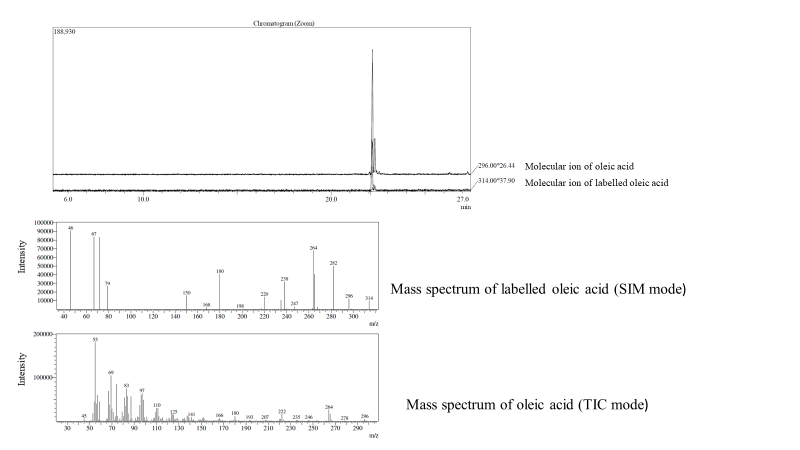
Supplementary Figure 1 – chromatogram and mass spectra of native and radiolabelled stearic acid (A) and oleic acid (B).

**B**

**A**
